# Cytoprotective Potential of Aged Garlic Extract (AGE) and Its Active Constituent, S-allyl-l-cysteine, in Presence of Carvedilol during Isoproterenol-Induced Myocardial Disturbance and Metabolic Derangements in Rats

**DOI:** 10.3390/molecules26113203

**Published:** 2021-05-27

**Authors:** Syed Mohammed Basheeruddin Asdaq, Obulesu Challa, Abdulhakeem S. Alamri, Walaa F. Alsanie, Majid Alhomrani, Abdulrahman Hadi Almutiri, Majed Sadun Alshammari

**Affiliations:** 1 Department of Pharmacy Practice, College of Pharmacy, AlMaarefa University, Dariyah, Riyadh 13713, Saudi Arabia; 2 Department of Pharmacology, Krupanidhi College of Pharmacy, Bangalore 560035, India; emadfaiqa@gmail.com; 3 Department of Clinical Laboratory Sciences, The Faculty of Applied Medical Sciences, Taif University, Taif 21944, Saudi Arabia; a.alamri@tu.edu.sa (A.S.A.); w.alsanie@tu.edu.sa (W.F.A.); m.alhomrani@tu.edu.sa (M.A.); 4 Centre of Biomedical Sciences Research (CBSR), Deanship of Scientific Research, Taif University, Taif 21944, Saudi Arabia; 5 Ministry of Health, Cluster one Riyadh, King Salman Hospital, Riyadh 12769, Saudi Arabia; abhalmutiri@moh.gov.sa; 6 King Abdulaziz Medical City in Riyadh, Ministry of National Guard, Riyadh 14611, Saudi Arabia; Majedalhosaini@gmail.com

**Keywords:** aged garlic extract, S-allyl cysteine, isoproterenol, myocardial disturbance, metabolic derangements, carvedilol, cytoprotection

## Abstract

This study was conducted to determine the potential interaction of aged garlic extract (AGE) with carvedilol (CAR), as well as to investigate the role of S-allyl-l-cysteine (SAC), an active constituent of AGE, in rats with isoproterenol (ISO)-induced myocardial dysfunction. At the end of three weeks of treatment with AGE (2 and 5 mL/kg) or SAC (13.1 and 32.76 mg/kg), either alone or along with CAR (10 mg/kg) in the respective groups of animals, ISO was administered subcutaneously to induce myocardial damage. Myocardial infarction (MI) diagnostic predictor enzymes, lactate dehydrogenase (LDH) and creatinine kinase (CK-MB), were measured in both serum and heart tissue homogenates (HTH). Superoxide dismutase (SOD), catalase, and thiobarbituric acid reactive species (TBARS) were estimated in HTH. When compared with other groups, the combined therapy of high doses of AGE and SAC given alone or together with CAR caused a significant decrease in serum LDH and CK-MB activities. Further, significant rise in the LDH and CK-MB activities in HTH was noticed in the combined groups of AGE and SAC with CAR. It was also observed that both doses of AGE and SAC significantly increased endogenous antioxidants in HTH. Furthermore, histopathological observations corroborated the biochemical findings. The cytoprotective potential of SAC and AGE were dose-dependent, and SAC was more potent than AGE. The protection offered by aged garlic may be attributed to SAC. Overall, the results indicated that a high dose of AGE and its constituent SAC, when combined with carvedilol, has a synergistic effect in preventing morphological and physiological changes in the myocardium during ISO-induced myocardial damage.

## 1. Introduction

Simultaneous administration of herbs and drugs has the potential to imitate, magnify, or oppose each of their individual pharmacological effects [1]. While herbs show promise as therapeutically effective medicines, it is generally believed that further research is needed to validate their effectiveness when given along with conventional medications.

Garlic (*Allium sativum*) has been used as a flavoring ingredient, functional food, and conservative remedy for thousands of years. The strong relationship between dietary patterns, including garlic consumption, and the incidence of disease has been demonstrated in epidemiological, clinical, and preclinical studies [2]. A large number of research articles have been published in the last few decades that demonstrate the therapeutic benefits of garlic, so it is widely regarded as a food item with strong and diverse benefits. Commercially, a number of garlic preparations are available as homogenate, oil, powder, and liquid/dried extracts. These preparations have a wide range of chemical compositions. Unique constituents present in garlic and its extracts are thought to be responsible for the medicinal and beneficial properties [3].

While there are several commercially available garlic preparations, there is still some confusion due to the uncertainty of clinical study findings and the lack of scientific research on individual products. Different methods of garlic preparation contribute to the activation of specific bioactive constituents, which may explain the contradictory efficacies. According to a previous study, garlic homogenate (GH)-mediated myocardium protection was attributed to the active organosulfur metabolites S-allylcysteine (SAC) and S-allylmercaptocysteine (SAMC), both of which have antioxidant potential [4]. Previously, it was assumed that allicin (allyl 2-propenethiosulfinate) was the most biologically active compound responsible for the cardioprotective effect. Subsequently, it is now clear that allicin is only a transient substance with oxidant property that is not detectable in blood, and it spontaneously gets converted into SAC and SAMC [5].

Aged garlic extract (AGE) is a garlic supplement that is less unpleasant and does not have the same negative effects as garlic [6]. Several published studies documented the antioxidant, anti-cancer, and cardio/hepato-protective potential of AGE and its active constituents [7,8,9,10]. S-allyl-l-cysteine, S-methyl-l-cysteine, S-ethylcysteine, S-1-proponyl-l-cysteine, S-allylmercapto-l-cysteine, fructosyl-arginine, beta-chlorogenin, l-arginine, l-cysteine, and l-methionine are among the constituents of AGE [9]. S-allylcysteine is the most active component of AGE (SAC) [11]. It has been shown to have antioxidative, anti-cancer, and anti-hepatotoxic [12] properties. Due to its antioxidative potential, which suppresses lipid peroxidation products, SAC provides protection to the myocardium during cardiac injury [13].

Carvedilol is a one-of-a-kind multi-action drug that is a nonselective beta blocker with additional vasodilating properties due to an α_1_ blockade [14]. It functions as an antianginal [15], antihypertensive [16], antiarrhythmic [17], antiproliferative [18], and potent antioxidant [19]. Carvedilol has been shown in previous studies to help patients with both idiopathic and ischemic congestive heart failure [20,21], as well as patients with left ventricular dysfunction [22]. Furthermore, a study published recently reported the significant mortality benefits of combining carvedilol with standard care in patients with congestive heart failure (65%) [23]. In addition, carvedilol ameliorates hyperlipidemia [24]. Moreover, it is shown to reduce the infarct size [25] and to obviate renal dysfunction [26] in animal models.

As previously stated, garlic has the great benefit of protecting the myocardium during times of stress [27], and carvedilol is a potent cardioprotective agent; however, because of their ability to protect the myocardium from injurious agents, it would be interesting to learn more about the function of combination therapy of potent garlic preparations, aged garlic extract (AGE), and carvedilol during myocardial damage. It was also our aim to estimate the quantity of the most effective sulphur-containing substances of AGE, s-allyl cysteine (SAC), and thereby to explore in rats the influence of SAC during isoproterenol-induced myocardial disturbance and metabolic derangement in the presence and absence of carvedilol.

## 2. Results

### 2.1. HPTLC Profile of S-allyl-l-cysteine and Aged Garlic Extract (AGE)

**Figure 1:**
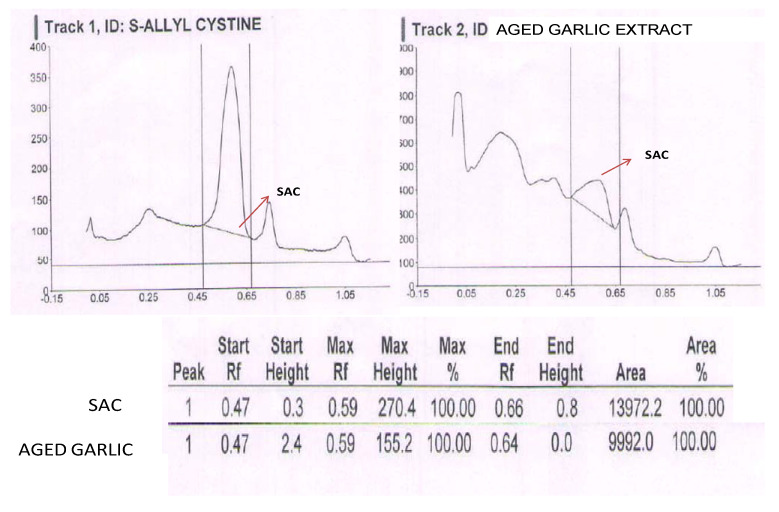
Typical chromatogram of standard S-allyl cysteine (SAC) and aged garlic extract.

The HPTLC chromatogram of aged garlic extract (AGE) and S-allyl cysteine (SAC) is shown in Figure 1. The assay of SAC in AGE extract was found to be 0.56%.

### 2.2. Effect on Hemodynamic Parameters

#### 2.2.1. Effect on Lactate Dehydrogenase Activity

**Figure 2:**
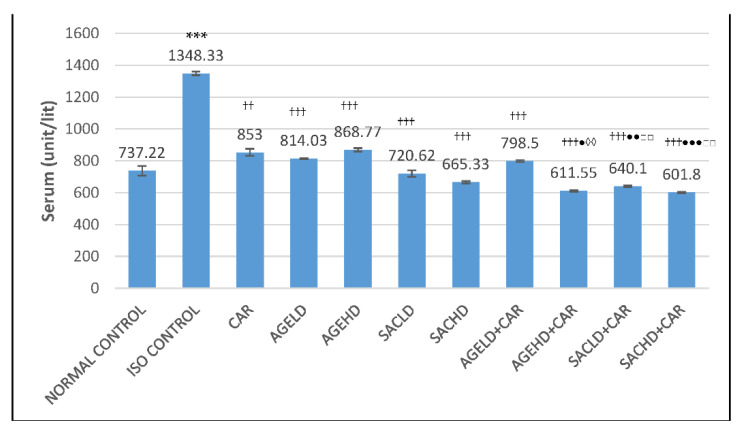
Effect on serum LDH activities. The data are given as mean ± SEM; *** *p* < 0.001 compared with normal control; ^††^ *p* < 0.01, ^†††^ *p* < 0.001 in comparison with ISO control; ^●^ *p* < 0.05, ^●●^ *p* < 0.01, ^●●●^ *p* < 0.001 in comparison with carvedilol; ^◊◊^ *p* < 0.01, when compared with respective AGE dose; ^□□^ *p* < 0.01 when compared with respective SAC dose; ISO (isoproterenol, 150 mg/kg); CAR (carvedilol 10 mg/kg); AGELD (aged garlic extract low dose 2 mL/kg); AGEHD (aged garlic extract high dose 5 mL/kg); SACLD (S-allyl cysteine low dose 13.1 mg/kg); SACHD (S-allyl cysteine high dose 32.76 mg/kg).

**Figure 3:**
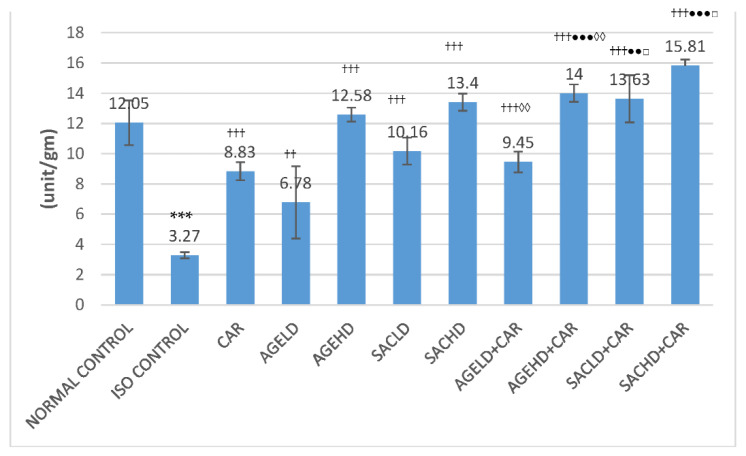
Effect on LDH activities in heart tissue homogenate. The data are presented as mean ± SEM; *** *p* < 0.001 compared with normal control; ^††^ *p* < 0.01, ^†††^ *p* < 0.001 in comparison with ISO control; ^●●^ *p* < 0.01, ^●●●^ *p* < 0.001 in comparison with carvedilol; ^◊◊^ *p* < 0.01 when compared with respective AGE dose; ^□^ *p* < 0.05 when compared with respective SAC dose.

Administration of two doses of isoproterenol (ISO) produced a significant reduction in the LDH activity in heart tissue homogenate (HTH) and a rise in serum LDH (lactate dehydrogenase) activity in comparison with the normal control group. When compared with ISO, all treated groups displayed a significant decrease in serum LDH levels (Figure 2). Groups treated with SACHD (S-allyl cysteine high dose) and AGEHD (aged garlic extract high dose) concurrently with CAR (carvedilol) demonstrated a significant reduction in LDH activity in serum when compared with the CAR cohort alone. Concurrent administration of SAC with CAR showed significant alteration in LDH level when compared with SAC alone. Additionally, AGE with CAR caused a significant increase in LDH level in HTH compared with the AGE alone group (Figure 3).

#### 2.2.2. Effect on Creatine Kinase-MB Activity

**Figure 4:**
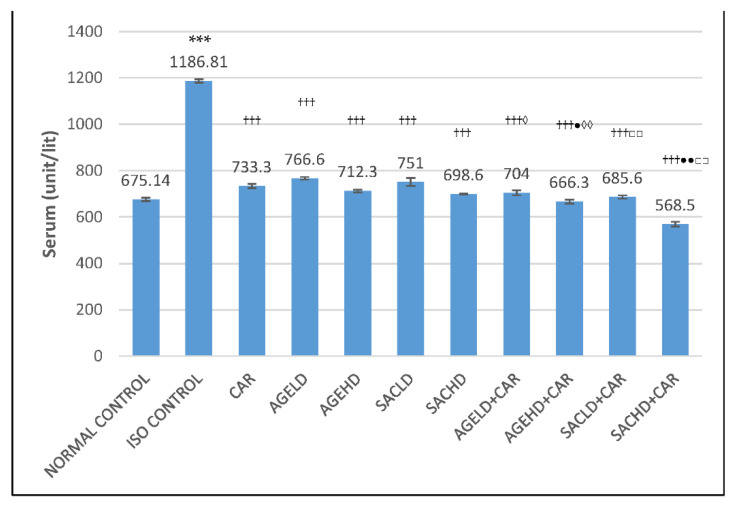
Effect on serum CK-MB activities. The data are presented as mean ± SEM; *** *p* <0.001 compared with normal control; ^†††^ *p* < 0.001 in comparison with ISO control; ^●^ *p* < 0.05, ^●●^ *p* < 0.01 in comparison with carvedilol; ^◊^ *p* < 0.05, ^◊◊^ *p* < 0.01 when compared with respective AGE dose; ^□□^ *p* < 0.01 when compared with respective SAC dose.

**Figure 5:**
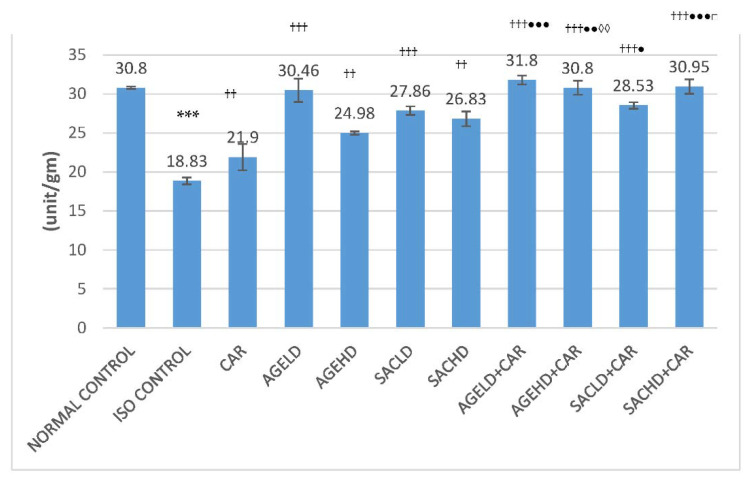
Effect on CK-MB activities in heart tissue homogenate. The data are given as mean ± SEM; *** *p* < 0.001 compared with normal control; ^††^ *p* < 0.01, ^†††^ *p* < 0.001 in comparison with ISO control; ^●^ *p* < 0.05, ^●●^ *p* < 0.01, ^●●●^ *p* < 0.001 in comparison with carvedilol; ^◊◊^ *p* < 0.01 when compared with respective AGE dose; ^□^ *p* < 0.05 when compared with respective SAC dose.

In the ISO control group, creatine kinase-MB (CK-MB) activities in serum were significantly elevated, while a decrease in these enzyme activities was observed in HTH of the same animals when compared with the normal control group. When compared with ISO, all treated groups displayed a significant reduction in serum CK-MB activities (Figure 4). AGEHD + CAR and SACHD + CAR groups exhibited a significant decrease in CK-MB activities in serum in comparison with CAR-treated animals. Similarly, the AGEHD + CAR, SACLD + CAR, and SACHD + CAR groups demonstrated an increase in CK-MB activities when compared with the CAR alone group. Additionally, both low and high doses of SAC and AGE in combination with CAR exhibited significant alteration in the CK-MB activities in comparison with the animals that received only SAC (Figure 5).

#### 2.2.3. Effect on Superoxide Dismutase and Catalase

**Figure 6:**
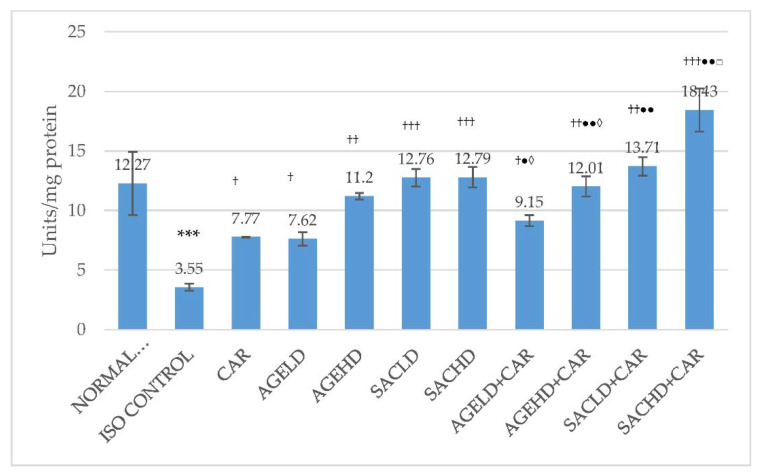
Effect on superoxide dismutase activities. The data are given as mean ± SEM; *** *p* < 0.001 compared with normal control; ^†^ *p* < 0.05, ^††^ *p* < 0.01, ^†††^ *p* < 0.001 in comparison with ISO control; ^●^ *p* < 0.05, ^●●^ *p* < 0.01 in comparison with carvedilol; ^◊^ *p* < 0.05 when compared with respective AGE dose; ^□^ *p* < 0.05 when compared with respective SAC dose.

**Figure 7:**
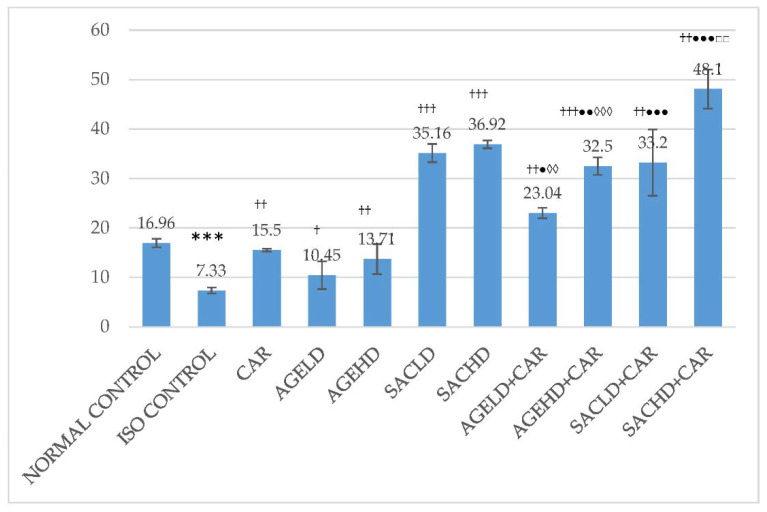
Effect on catalase activities. The data are given as mean ± SEM; *** *p* < 0.001 compared with normal control; ^†^ *p* < 0.05, ^††^ *p* < 0.01, ^†††^ *p* < 0.001 in comparison with ISO control; ^●^ *p* < 0.05, ^●●^ *p* < 0.01, ^●●●^ *p* < 0.001 in comparison with carvedilol; ^◊◊^ *p* < 0.01, ^◊◊◊^ *p* < 0.001 when compared with respective AGE dose; ^□□^ *p* < 0.01 when compared with respective SAC dose.

Two days of injection of ISO resulted in a reduction in SOD and catalase activities in HTH. ISO administration produced a significant reduction in the SOD and catalase activities in comparison with normal control. Further, all treated groups displayed a significant rise in these enzyme activities compared with ISO control. Significantly increased activities were observed in groups that received AGE and SAC along with CAR when compared with groups that received only CAR (Figure 6 and Figure 7). AGE low and high doses with CAR showed significantly better activities of SOD and catalase when compared with groups that received AGE and SAC, respectively.

#### 2.2.4. Effect on Thiobarbituric Acid Reactive Species

**Figure 8:**
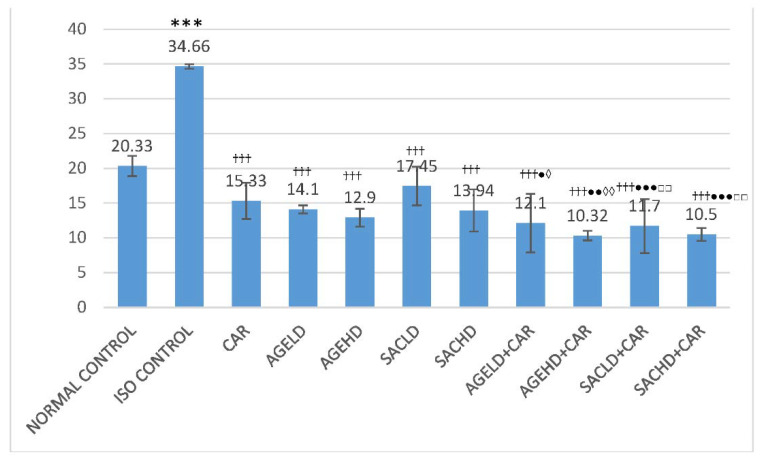
Effect on thiobarbituric acid reactive species. The data are given as mean ± SEM; *** *p* < 0.001 compared with normal control; ^†††^ *p* < 0.001 in comparison with ISO control; ^●^ *p* < 0.05, ^●●^ *p* < 0.01, ^●●^ *p* < 0.001 in comparison with carvedilol; ^◊^ *p* < 0.05, ^◊◊^ *p* < 0.01 when compared with respective AGE dose; ^□□^ *p* < 0.01 when compared with respective SAC dose.

When ISO was given, the levels of thiobarbituric acid reactive species (TBARS) significantly increased when compared with normal. The HTH of animals that received either AGE, SAC alone, or along with CAR before being subjected to ISO administration showed a significant decrease in TBARS as compared with ISO control. Additionally, groups that were given AGE or SAC with CAR exhibited significantly low level of TBARS when compared with CAR alone. Furthermore, a significantly decreased level of TBARS was observed in groups that received AGE and SAC along with CAR compared with groups that were pretreated with respective doses of AGE and SAC (Figure 8).

### 2.3. Histological Investigations

**Figure 9:**
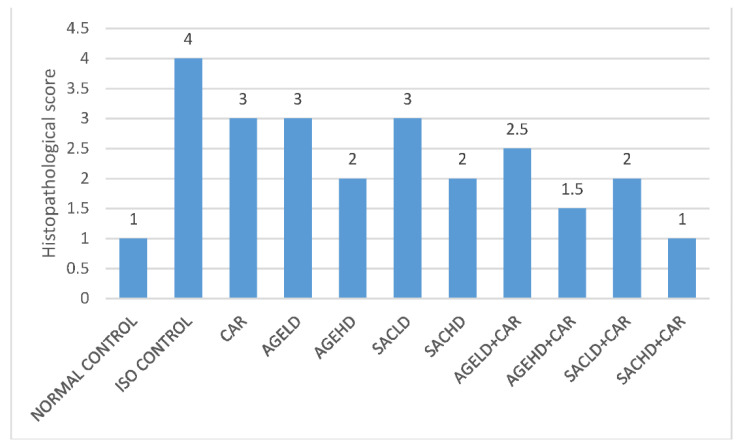
Effect on histopathological score.

The histopathological score of ISO-treated animals was high when compared with all other groups (Figure 9). A high dose of SAC in the presence of CAR showed normal musculature of cardiac cells, which is similar to normal animals’ myocardium.

**Figure 10:**
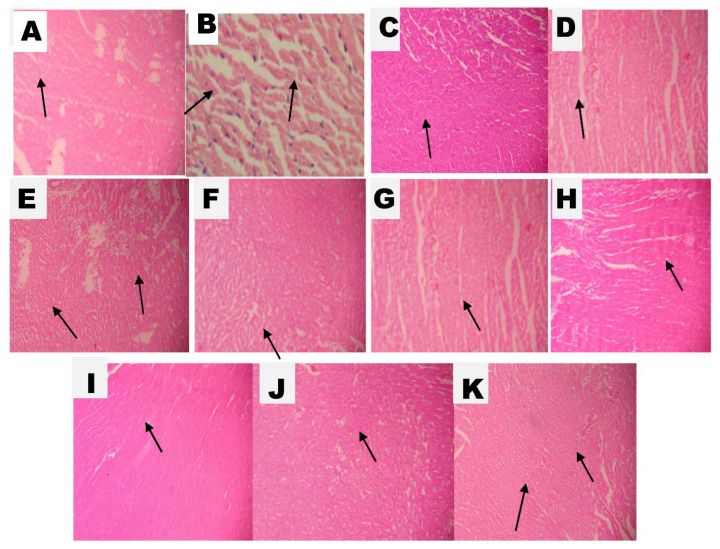
Hematoxylin and eosin (**H**&**E**) stained section of hearts of different study groups. Photographed at magnification 400×. (**A**) Normal control group shows normal myocardial structure with intact cell membrane without necrosis, (**B**) ISO control shows vacuolar change and necrosis with focal cell infiltration with inflammatory patches, (**C**) carvedilol-treated groups (10 mg/kg) with intact architecture, (**D**) AGE low-dose-treated group (2 mL/kg) with slight increase in the intercellular space and decrease in the size of the cardiocytes in some areas, (**E**) AGE high dose (5 mL/kg) with some areas showing haphazardly arranged cardiocytes, (**F**) SAC low dose (13.1 mg/kg) shows patchy areas of mild necrosis, (**G**) SAC high dose (32.76 mg/kg) shows mild decreased size of some cardiocytes, (**H**) AGE low dose + CAR shows moderately diffused necrosis, (**I**) AGE high dose + CAR, (**J**) SAC low dose + CAR with moderately diffused necrosis and inflammatory patches, and (**K**) SAC high dose + CAR shows almost normal cardiac musculature.

The vehicle-treated group, the AGEHD + CAR group, and the SACHD + CAR group all had minimal necrosis (Figure 9). The ISO group had severe necrosis, inflammation, and fibrosis. The CAR and AGELD treatment groups had moderately diffused necrosis, slight inflammation, and fibrosis. In the AGEHD, SACHD, AGELD + CAR, AGEHD + CAR, and SACLD + CAR groups, mild diffuse necrosis, moderate necrosis, and mild inflammation were observed (Figure 10).

## 3. Discussion

The therapeutic benefits of several types of garlic preparations have been extensively investigated, and each preparation has a variety of active constituents that decompose and turn into a range of therapeutically active biological compounds. In this study, we determined the cytoprotective potential of one therapeutically effective garlic preparation, aged garlic extract and its active constituent s-allyl cysteine (SAC), in combination with carvedilol during myocardial dysfunction and metabolic derangements caused by isoproterenol in rats.

Catecholamines are important regulators of myocardial contractility and metabolism. However, excess catecholamines is associated with cellular disruption, as shown by angina, transient myocardial hypoxia, acute coronary insufficiency, and subendocardial infarction. Isoproterenol, a powerful synthetic catecholamine, when given to animals, develops infarct-like lesions [28]. Isoproterenol (ISO), an adrenergic agonist, was used to cause myocardial infarction in this study. This is a standard model for investigating the effectiveness of novel myocardial cytoprotective agents. Within 48 h of receiving ISO, changes in myocardial integrity, cellular disruption, and biochemical changes are widely reported [2]. Free radicals are generated when catecholamines are spontaneously oxidized (redox reactions), and the oxidized products may interact with the sulphydryl groups of various proteins, causing superoxide anion production that further transforms into hydrogen peroxide [29].

Several traditional medical systems have long found garlic (*Allium sativum* L.) to be a beneficial curing agent. At least two different forms of garlic are commercially recognized: those that contain allicin and those without allicin. The former is made with raw garlic, while the latter is made with “processed” garlic. The substances they contain can differ significantly [30]. Garlic organosulfur compounds (OSCs) are a relatively new class of chemopreventive compounds [31]. Garlic OSCs have been shown to influence drug metabolism systems, especially phase II detoxifying enzymes [32].

Carvedilol is a relatively new anti-anginal, anti-arrhythmic, and antihypertensive medication. It is also used to treat ischemic and idiopathic congestive heart failure. The exact mechanism underlying the beneficial hemodynamic effects of β-adrenoceptor blockers is unknown. In the case of carvedilol, β1-adrenoceptors can have beneficial effects by lowering heart rate and ameliorating the harmful effects of norepinephrine on the myocardium. Because of its potential function in the presynaptic modulation of catecholamine release [33], the existence of β2-adrenoceptor antagonism may be significant. Carvedilol also has antioxidant properties, but the significance of this property is unknown.

The “aging method” extracts AGE from garlic cloves after an extensive extraction process. The aging causes complete hydrolysis of g-glutamyl cysteines to S-allyl cysteine (SAC) and S-1-propenyl cysteine. Additionally, it results in an increase in cysteine (due to protein hydrolysis) and S-allyl mercapto cysteine [34,35]. Further, we used a standard chromatographic technique [36], high-performance thin-layer chromatography (HPTLC), to identify and quantify SAC in AGE.

The cardiac diagnostic enzymes, LDH and CK-MB, liberate out of the myocardial cells into blood circulation due to myocardial injury. The volume of these enzymes in the serum is an indicator of the extent of damage to cardiac cells [37]. In the current research, isoproterenol-injected rats had significantly higher serum levels of these enzymes, confirming damage to the myocardium that was consistent with an earlier study [38]. When AGE and SAC were given together with carvedilol, the activity of the marker enzymes was reduced in serum and increased in heart homogenate, suggesting that AGE/SAC has a greater cardioprotective capacity in the presence of carvedilol.

In the heart tissues of isoproterenol-injected animals, antiperoxidative enzyme activities (SOD and catalase) was substantially reduced. Reduced antiperoxidative enzyme activities result in less removal of superoxide and hydrogen peroxide radicals. SOD and catalase levels in heart tissue were restored after pretreatment with AGEHD + CAR and SACHD + CAR. Carvedilol’s effect could be attributable to a combination of beta-blocking and antioxidant potential (36), while AGE and SAC’s effect could be due to their ability to enhance antioxidant formation [39]. Peroxidation converts membrane phospholipids, specifically esterified polyunsaturated fatty acids, to MDA, which can be measured by thiobarbituric acid reactivity [40]. TBARS (thiobarbituric acid reactive substances) are a direct indicator of cell lipoperoxidation. TBARS levels in the treated groups were significantly reduced due to the free radical scavenging ability of AGE and its active constituent, SAC. Additionally, concurrent administration of carvedilol, a recognized free radical scavenger [8] with SAC and AGE significantly reduced free radical development.

Histopathological scores revealed and documented damage to the cardiac musculature. Myocardial damage is indicated by an increase in score [41]. When compared with the ISO-treated group, pretreatment with high doses of AGE and SAC alone or with carvedilol reduced pathological scores and preserved myocardial integrity during ISO injury. SAC at high doses in combination with CAR resulted in a lower histopathological score, indicating cardioprotection. This effect may be due to an increase in the production of endogenous antioxidant enzymes.

Many of the common uses of garlic in medicine have been confirmed owing to studies on the effects of AGE. The high content of stable and highly bioavailable water-soluble organosulfur compounds in AGE contributes to its health benefits and high antioxidant activity as compared with other commercial preparations. It has been proposed that AGE improves vascular endothelial function and myocardial integrity [42], both of which are essential in myocardial dysfunction and metabolic disorders. Further, the most active constituent of AGE, SAC, maintained the integrity of the mitochondrial and lysosomal membranes and restored the enzyme activities to near-normal levels. Both AGE and its active constituent, SAC, demonstrated these cardioprotective benefits by virtue of their antioxidant potentials [43]. Nonetheless, as a stand-alone or adjunctive drug, aged garlic extract and its active constituent, SAC, has shown promising results as a cardioprotective agent.

## 4. Materials and Methods

### 4.1. Experimental Animals

Female Sprague-Dawley rats weighing 150–200 g were housed at 25 ± 5 °C in a well-ventilated animal house with a 12:12 h light-dark cycle. The experimental procedure was accepted by the Institutional Animal Ethics Committee (KCP/IAEC-27/08-09). The animals were kept in an animal house under normal conditions, as directed by the Committee for the Purpose of Control and Supervision of Experiments on Animals (CPCSEA).

### 4.2. Preparation of Aged Garlic Extract (AGE)

Fresh garlic was extracted in 25% ethanol for a duration of 10 months to produce AGE at ambient temperature, and the extract was stored at 4 °C [44]. After that, the AGE was decanted using muslin cloth. The clear solution was filtered through Whatman No.1 filter paper after decanting the extract and storing it at 20 °C before use [7].

### 4.3. Apparatus and Chemical Used

B&K Technology Group China Co., Ltd. provided a standard SAC sample (Xiamen, China). The CK-MB and LDH kits were provided by Crest Biosystems and Coral Clinical Systems in Goa, India. The DL-isoproterenol hydrochloride was given by Sigma Aldrich in the United States. Nice Chemicals Pvt Ltd. in Cochin, India, provided the EDTA, and Hong Yang Chemical Corp. in China provided the ethanol. Heparin was provided by Gland Pharmaceutical Ltd. in Hyderabad, India. Hydrogen peroxide, hydroxylamine HCL, and ketamine were provided by Prem Pharmaceutical Ltd. Indore, India. Malondialdehyde (MDA), n-Butanol, and nitro blue tetrazolium were supplied by Loba Chemicals in Mumbai, India (NBT). Standard bovine albumin and sucrose were provided by S D Fine Chemicals in Mumbai, India. The thiobarbituric acid and xylazine were provided by Indian Immunological in Guntur, India. Copper sulphate, disodium hydrogen orthophosphate, phenol reagent, phosphoric acid, potassium dihydrogen orthophosphate, sodium chloride, sodium hydroxide, and sodium carbonate were given by Merck Specialties Private Limited in Mumbai, India. All of the other chemicals used in this experiment were obtained from well-known suppliers. Qualigens in Mumbai, India, and INCO in India sold AutoAnalyzer. Before being used, all equipment and instruments used in this study were calibrated.

### 4.4. High-Performance Thin-Layer Chromatography (HPTLC) Analysis of AGE

A high-performance thin-layer chromatographic technique was used to identify and quantify SAC in AGE. A portion of 2 mg of S-allyl cysteine was dissolved in 10 mL of filtered methanol and concentrated to 5 mL to prepare standard preparation, whereas sample was prepared by extracting 250 mg of aged garlic with 10 m methanol for 30 min. The aged garlic sample was filtered and concentrated to 5 mL [45]. A CAMAG HPTLC device (including a Linomat-5 applicator, a Digistore-2 multiwavelength scanner, a transparent chromatographic tank, an HPTLC pre-coated silica tray, and a silica gel 60 F254, 10 × 10 cm (Merck, Kenilworth, NJ, USA)) was used to assess SAC and AGE. Both the injector and the detector were set to 60 degrees Celsius. The mobile phase consisted of ethyl acetate, acetic acid, water, and acetone, (9:1:1:1), and the detector was ninhydrin. The region due to SAC in the chromatogram was defined after a volume of 20 μL of standard, and sample solutions were injected, and the amount of SAC in the AGE sample was calculated using the standard formula.

(STD wt/Sample wt) × (Sample area/STD area) × assay of STD = Assay of S-allyl cysteine.

### 4.5. Selection of Doses

Carvedilol (10 mg/kg) [28] and aged garlic extract (2/5 mL/kg) [46] doses were used based on previous studies. The SAC HPTLC peaks in the AGE spectra led the measurement of SAC doses (13.1/32.76 mg/kg). The two SAC doses were chosen based on a 2:5 ratio, with 13.1 and 32.76 mg of SAC present in 2 and 5 mL/kg of AGE, respectively.

### 4.6. Experimental Protocol

Animals used in this study were divided into eleven groups (n = 6). Group I and II received placebo treatment and Isoproterenol (150 mg/kg, s.c.), respectively. The third group was given carvedilol (10 mg/kg), whereas group IV, V, VI, and VII were administered with AGE low dose (2 mL/kg), AGE high dose (5 mL/kg), SAC low dose (13.1 mg/kg), and SAC high dose (32.76 mg/kg), respectively. The eighth, ninth, tenth, and eleventh groups received carvedilol along with AGE low dose, AGE high dose, SAC low dose, and SAC high dose, respectively. All treatments were given for three weeks.

### 4.7. Isoproterenol-Induced Myocardial Necrosis in Rats

Animals in all groups, with the exception of the standard control, were given ISO (150 mg/kg, s.c.) for two days after completing their respective treatments. Rats were given a mixture of ketamine hydrochloride (75 mg/kg, i.p.) [47] and xylazine (10 mg/kg, i.p.) [48] 48 h after receiving their first dose of ISO [27]. LDH, CK-MB, and thiobarbituric acid reactive species (TBARS) were determined using separated serum of the blood samples [49]. Three hearts from each group were removed and homogenized with sucrose to prepare heart tissue homogenate (HTH) (0.25 M). Endogenous biological markers such as LDH, CK-MB, and antioxidants including superoxide dismutase (SOD) [50] and catalase [51] were measured in HTH. The remaining three hearts were used for the preparation of microscopic slides and subjected to stopathological and electron microscopic examinations.

### 4.8. Preparation of Heart Tissue Homogenate

With the help of scissors, the hearts were freed of the surrounding vessels and fatty tissue mass after the experiment [51]. After that, the hearts were sliced open, rinsed with saline (0.9 percent NaCl), and dried with filter paper. Using a mortar and pestle, the heart was weighed and homogenized in an ice cold 0.25 M sucrose solution. The homogenate was centrifuged for 15 min at 5000 rpm. The supernatant was decanted and used to measure CK-MB, LDH, SOD, catalase, and TBARS concentrations.

### 4.9. Histopathological Examination

The heart tissues from all groups were immediately washed in saline and then fixed in a 10% *v*/*v* formalin in saline solution. The left ventricular mass was removed from the core to obtain a 0.4 cm thick cross-over section, which was then dried with alcohol before being embedded in paraffin wax. Hematoxylin and eosin (H&E) were used to stain these sections [52]. The damage to the myocardium was evaluated on the basis of the extent of damage to the cardiac cells. No change in the myocardial integrity was scored 00, whereas the score 01 was given to the myocardium with central myocyte damage or little multifocal degeneration with a mild inflammatory process level. The score 02 was assigned to myocardium with broad myofibrillar degeneration or a potentially diffuse inflammatory process, while the high score of 03 was given to the cardiac cells having diffused inflammatory necrosis [53].

### 4.10. Statistical Analysis

To assess statistical significance, one-way analysis of variance (ANOVA) was used [27], followed by Dunnett’s comparison tests using the GraphPad Prism 8.0 computer software kit. The results are expressed as mean and SEM, with a significance level of *p* < 0.05 assumed.

## 5. Conclusions

To summarize, both AGE and SAC offer strong cytoprotection to myocardium, and they work in tandem with carvedilol to improve its cardioprotective properties. The findings indicate that combining AGE or SAC with carvedilol may protect susceptible patients from myocardial injury more effectively than carvedilol alone. Since the toxic effects of these combinations were not assessed in this study, more toxicological research is required before concluding that carvedilol in combination with AGE or SAC has a beneficial effect.

## Data Availability

Data are contained within the article.

## Sample Availability

Samples of the compounds are available from the authors.
